# Artificial intelligence in personalized nutrition and food manufacturing: a comprehensive review of methods, applications, and future directions

**DOI:** 10.3389/fnut.2025.1636980

**Published:** 2025-07-23

**Authors:** Kushagra Agrawal, Polat Goktas, Navneet Kumar, Man-Fai Leung

**Affiliations:** ^1^School of Computer Engineering, KIIT Deemed to be University, Bhubaneswar, India; ^2^UCD School of Computer Science, University College Dublin, Dublin, Ireland; ^3^ESM Division, ICAR - National Academy of Agricultural Research Management, Hyderabad, India; ^4^School of Computing and Information Science, Faculty of Science and Engineering, Anglia Ruskin University, Cambridge, United Kingdom

**Keywords:** artificial intelligence, personalized nutrition, food manufacturing, machine learning, federated learning, predictive analytics

## Abstract

Artificial Intelligence (AI) is emerging as a key driver at the intersection of nutrition and food systems, offering scalable solutions for precision health, smart manufacturing, and sustainable development. This study aims to present a comprehensive review of AI-driven innovations that enable precision nutrition through real-time dietary recommendations, meal planning informed by individual biological markers (*e.g*., blood glucose or cholesterol levels), and adaptive feedback systems. It further examines the integration of AI technologies in food production, such as machine learning–based quality control, predictive maintenance, and waste minimization, to support circular economy goals and enhance food system resilience. Drawing on advances in deep learning, federated learning, and computer vision, the review outlines how AI transforms static, population-level dietary models into dynamic, data-informed frameworks tailored to individual needs. The paper also addresses critical challenges related to algorithmic transparency, data privacy, and equitable access, and proposes actionable pathways for ethical and scalable implementation. By bridging healthcare, nutrition, and industrial domains, this study offers a forward-looking roadmap for leveraging AI to build intelligent, inclusive, and sustainable food–health ecosystems.

## 1 Introduction

With nutrition-related chronic conditions such as obesity, diabetes, and cardiovascular diseases on the rise, there is a growing imperative to shift from generalized dietary guidelines toward individualized, data-driven nutritional strategies. While the importance of optimal nutrition in health promotion and disease prevention is well-established, traditional dietary planning often relies on generalized frameworks that overlook inter-individual variability (1, 2). These static, population-level guidelines are insufficient to address the complex interplay of genetics, metabolic markers, lifestyle behaviors, and environmental exposures that influence nutritional needs. Consequently, a significant proportion of individuals receive dietary recommendations that fail to produce the intended health benefits. At the same time, the food manufacturing sector faces mounting scrutiny over issues related to nutrient degradation, lack of transparency, and limited adaptability in production processes. Conventional manufacturing practices frequently compromise the nutritional quality of food products, while insufficient traceability in the food supply chain raises concerns over safety and nutritional reliability (3). These dual challenges, generic dietary guidance and inefficient food production systems, necessitate a paradigm shift driven by technological innovation.

Artificial intelligence (AI) has emerged as a transformative enabler in this context. Through advanced techniques such as machine learning (ML) and deep learning (DL), AI facilitates the extraction of actionable insights from complex health and dietary datasets (4, 5). AI-powered systems are increasingly capable of delivering real-time, individualized dietary recommendations, especially for chronic disease management. Continuous glucose monitoring platforms, for example, leverage AI algorithms to support personalized dietary decisions for diabetic patients, fostering better glycemic control and improved outcomes (6). Similarly, AI-enabled remote patient monitoring systems dynamically adjust nutritional recommendations based on ongoing physiological changes, offering a responsive approach to dietary management (7).

Beyond personalized nutrition, AI contributes significantly to enhancing the food manufacturing process. Tools such as artificial neural networks and fuzzy logic models have been applied to optimize drying technologies, enabling manufacturers to better preserve nutritional content during processing (1, 8). AI also supports the production of customized food formulations to meet specific dietary needs, contributing to the broader goal of precision nutrition. Furthermore, AI-driven traceability systems improve transparency and monitoring across the supply chain, ensuring that food quality is maintained from source to consumer (3). However, the adoption of AI in nutrition and food science is not without challenges. Data privacy, security, and ethical concerns surrounding algorithmic decision-making are critical barriers that demand careful scrutiny (9). Additionally, long-term evidence on the efficacy, scalability, and societal impact of AI-based nutrition interventions remains limited, particularly across diverse populations and healthcare systems (1, 10). Limited explainability in complex AI models further complicates clinical and consumer trust, emphasizing the need for transparent, interpretable, and user-centered AI tools (2).

To address these critical gaps, this study aims to provide a comprehensive review of AI applications at the intersection of personalized nutrition and intelligent food manufacturing. By synthesizing current research and highlighting both opportunities and constraints, the paper contributes to advancing knowledge and practice in this evolving domain. Specifically, the study focuses on:

- Personalized dietary planning: exploring AI-driven methods for real-time, individualized nutrition strategies to support chronic disease management and preventive care.
- Food manufacturing innovation: investigating AI applications in food processing, including nutrient preservation, quality control, waste reduction, and resource optimization.
- Data privacy and security: assessing privacy-preserving AI approaches such as Federated Learning (FL) and homomorphic encryption for secure health data handling.
- Ethical and regulatory challenges: identifying the ethical dilemmas, interdisciplinary needs, and policy gaps associated with AI deployment in nutrition and food systems.
- Scalability and explainability: discussing the need for transparent, explainable AI models and scalable solutions across diverse populations and infrastructures.

By positioning AI at the intersection of personalized healthcare and intelligent food production, this study aims to advance research, support industry integration, and foster the development of ethical, resilient, and sustainable food–health ecosystems. To ensure a coherent and comprehensive analysis, the remainder of this paper is organized as follows: Section 2 examines AI-driven approaches to personalized dietary planning, focusing on ML and DL techniques. Section 3 discusses the use of predictive analytics to optimize health outcomes based on physiological and nutritional data. Section 4 explores the integration of AI in food manufacturing, with emphasis on quality control, sustainability, and process optimization. Section 5 highlights the importance of interdisciplinary collaboration among AI experts, nutritionists, and food technologists. Section 6 addresses ethical, regulatory, and societal challenges related to AI adoption in this domain. Finally, Section 7 summarizes key insights and outlines directions for future research to support responsible and impactful implementation of AI in nutrition and food systems.

## 2 AI in personalized nutrition: methods and applications

### 2.1 Defining personalized nutrition and its relevance in precision health

Personalized nutrition (PN) is defined as the adaptation of dietary recommendations based on individual-level variability in biology, behavior, and environment. It represents a shift from generalized nutritional guidance to precision-based approaches that accommodate genetic profiles, metabolic phenotypes, disease risks, and lifestyle patterns (11, 12). This paradigm is especially critical in addressing chronic conditions such as obesity, diabetes, and cardiovascular diseases, where standardized dietary interventions often fall short of achieving clinically meaningful outcomes (13).

Recent reviews emphasize that nutrigenomics, a field at the intersection of nutrition, genomics, and bioinformatics, forms a scientific foundation for PN by uncovering gene–nutrient interactions and enabling genotype-based dietary interventions (14). The integration of AI with nutrigenomics and multi-omics approaches has accelerated the implementation of PN strategies, providing more precise, individualized insights into dietary needs and health outcomes. For example, Waheed et al. (15) discuss how diet–gene interactions are crucial in managing neurological disorders such as Alzheimer's disease and Parkinson's disease. Their findings indicate that personalized diets guided by genetic insights and AI-assisted screening can significantly improve cognitive health. Similarly, Ferreira et al. (16) highlight how AI-enabled techniques such as random forests and gradient boosting enhance the prediction of individual responses to diets, particularly when microbiome data are included. These methods have demonstrated potential in managing weight, gastrointestinal health, and metabolic risks. Furthermore, Saha et al. (17) report that AI and computer vision driven automation in the food industry can achieve over 99% accuracy in food classification and nutrient detection. This high level of accuracy enables the real-time deployment of personalized dietary algorithms.

These advances point to a significant transformation in the PN landscape: a move from traditional heuristic-based dietary planning toward dynamic, data-driven frameworks powered by AI and supported by wearable biosensors, such as continuous glucose monitors (CGMs) and real-time nutrient trackers (18). As these technologies become increasingly integrated into health management platforms, AI-driven personalized nutrition is expected to play a crucial role in preventing disease, optimizing performance, and enabling long-term wellness strategies tailored to individual needs (19, 20).

### 2.2 Adaptive dietary planning with ML and reinforcement learning techniques

AI techniques, particularly ML and reinforcement learning (RL), have significantly advanced PN by enabling the integration and interpretation of complex, multimodal datasets. Supervised models such as multilayer perceptrons (MLPs) and long short-term memory (LSTM) networks have been employed to predict postprandial glycemic responses, lipid fluctuations, and weight dynamics, thereby transforming user-specific parameters into personalized, actionable dietary recommendations (21). Unsupervised methods like k-means clustering and principal component analysis (PCA) support phenotype-driven stratification for targeted interventions (22). Recent efforts have emphasized model transparency through symbolic knowledge extraction, facilitating explainable and rule-based recommendations aligned with expert guidance–demonstrated to reach 74% precision and 80% fidelity (23). RL algorithms, such as Deep Q-Networks and Policy Gradient methods, enable continuous personalization via feedback loops from behavioral and physiological data (e.g., CGM), reducing glycemic excursions by up to 40% (21, 24). Additionally, mobile health tools like Diet Engine have achieved 86% classification accuracy using DL (YOLOv8) for real-time food recognition and nutrient estimation (20).

Recent comprehensive reviews highlight recommender systems as a cornerstone in the field, often integrating wearable and app-based inputs (25). Hybrid models combining content-based filtering, collaborative algorithms, and knowledge graphs are increasingly adopted to enhance personalization and user wellbeing (26). In parallel, ML models such as random forests and XGBoost have been applied to biomarker prediction (e.g., plasma vitamin C), although limitations in data granularity remain (22). Despite promising outcomes, several implementation barriers persist, including ethical concerns related to autonomy and bias, variability in food databases, and the limited interpretability of deep models. Addressing these through explainable AI, robust validation, and clinical integration is essential for scalable and equitable PN applications (27, 28).

### 2.3 Image-based dietary assessment using DL and computer vision

Advancements in DL, especially convolutional neural networks (CNNs), have significantly enhanced the accuracy and efficiency of dietary assessment tools. These technologies automate tasks such as food image classification, portion size estimation, and nutrient content prediction, enabling more objective and scalable nutritional tracking. CNN-based models have consistently achieved classification accuracies above 85% across standard datasets (29, 30), and when paired with transformer-based architectures, such as CSWin or vision transformers, accuracy rates can exceed 90% in fine-grained food identification (31, 32).

A growing trend is the integration of attention mechanisms and multi-level feature fusion to improve recognition robustness in challenging conditions like intra-class similarity and variable lighting (30). Multi-level attention networks and knowledge distillation strategies have been shown to improve classification accuracy on large-scale datasets such as CNFOOD-241, a curated image dataset of Chinese food items with top-1 performance reaching 86.22% and top-5 accuracy up to 98.49% (32, 33). Similarly, ensemble-based models have leveraged both global context from transformers and local perception from CNNs to improve visual differentiation in complex food environments (33).

New frontiers include multimodal approaches that incorporate audio, text, and visual cues for enhanced summarization and dietary analysis. For example, transformer-based summarization models using GPT and Inception-V3 have been applied to cooking videos, extracting both visual ingredients and auditory recipe steps into structured meal records (34). Additionally, Multimodal Large Language Models are emerging in food energy estimation, incorporating reasoning capabilities and volume-aware inputs to improve caloric assessments (35). These innovations are not limited to academic development, real-world applications such as “*Diet Engine*” and mobile dietary assistants now employ YOLOv8-based CNN pipelines for real-time food recognition and nutrient estimation, achieving classification accuracy of 86% (20). Beyond image classification, AI systems now estimate the nutrient composition of complex dishes with a mean *R*^2^-top5 of 0.86, even for region-specific cuisines like Chinese dishes (33).

Despite these promising advances, several challenges persist, including the need for diverse, annotated food datasets, managing cross-cultural dietary differences, and ensuring model interpretability and generalizability across demographics. Table 1 summarizes the state-of-the-art models, datasets, and performance metrics associated with food image-based dietary assessment.

**Table 1:** Summary of deep learning-based models in image-based dietary assessment.

**Model/study**	**Key features and applications**	**Performance metrics**
FoodCSWin (29)	CSWin transformer with local feature dual enhancement block (LFDB); designed to manage large visual variance in food images	94.11% top-1 accuracy
MAF-Net (30)	Multi-level attention fusion using CNN backbones and KL-divergence regularization for fine-grained classification	90.61% (UEC Food-100)
ResVMamba (31)	Combines residual learning with selective state-space modeling; efficient for complex food image analysis in CNFOOD-241	81.70% top-1 accuracy
YOLOv8 – diet engine (20)	Mobile nutrition app using YOLOv8 and CNNs; supports image-based food recognition and chatbot-guided diet suggestions	86% classification accuracy
MLLM volume-aware model (35)	Multimodal large language model with volume-aware reasoning for improved caloric estimation from food images	Improved energy estimation accuracy on Nutrition5K
Ensemble CNN + transformer (32)	Uses ensemble learning and knowledge distillation to improve classification robustness and reduce model size	86.22% top-1 accuracy (Food2K)
RegNet fusion model (33)	Combines RegNet-Y with cutmix/mixup for nutrient estimation in Chinese cuisine; validated on CNFOOD-241 dataset	*R*^2^-Top5 = 0.8636
GPT + CNN video summarizer (34)	Automates cooking video summarization using CNN + GPT-based summarization pipeline; supports visual + audio synthesis	High qualitative accuracy in recipe extraction

### 2.4 Natural language processing for behavioral insights and digital dietary coaching

Natural Language Processing (NLP) plays an increasingly central role in capturing the behavioral dimensions of dietary assessment by analyzing text-based inputs such as food diaries, conversational logs with chatbots, and social media entries. Transformer-based architectures, including BERT and GPT models, have been deployed to extract patterns in eating behavior, detect anomalies (e.g., binge eating, late-night snacking), and assess emotional states influencing food choices (36, 37). One key application area is digital dietary coaching. Fadhil and Gabrielli (36) demonstrated that AI-based dietary chatbots significantly improved user adherence to nutrition plans by 32% over conventional counseling. This was attributed to AI's ability to offer continuous, context-aware, and emotionally adaptive feedback. Similarly, studies on multimodal journaling practices highlight users' varied preferences in food description strategies, ranging from vague portion sizes to detailed textual specifications, that challenge standard NLP pipelines (38).

Furthermore, Lan et al. (39) developed and evaluated “*iFood*,” a social-media-based applet designed for dietary monitoring. The system integrates food image recognition with NLP to interpret user-generated text from platforms like Weibo, demonstrating promising usability in real-world dietary logging. The study also highlighted the potential of combining visual and textual content for more accurate and user-friendly dietary monitoring. These findings indicate that combining multimodal NLP approaches with personalized feedback mechanisms offers a promising route for increasing user engagement, adherence, and effectiveness in digital nutrition programs. However, challenges remain in ensuring interpretability, cross-linguistic adaptability, and ethical considerations related to data sensitivity in user-generated content.

### 2.5 Clinical integration and consumer applications of AI-driven nutrition systems

AI-driven systems are increasingly integrated across clinical, consumer, and performance-based nutrition applications. In clinical contexts, explainable ML models such as support vector machines (SVMs) and random forests have demonstrated efficacy in identifying conditions like sarcopenic obesity using non-invasive, easily available features (e.g., body mass index, neck/thigh circumference), and are now supported by web-based tools for geriatric screening (40). Moreover, ChatGPT-generated dietary plans for metabolic dysfunction-associated steatotic liver disease (MASLD) show promising accuracy in caloric and fiber content, though improvements are needed in aligning macronutrient ratios with clinical guidelines (41).

Consumer-facing apps such as MyFitnessPal, Noom, and the WeChat-integrated iFood platform demonstrate how AI, combined with user-friendly interfaces and social media data, can promote self-tracking, adherence, and personalized dietary monitoring (39). NLP-powered tools like ChatGPT also show potential for multilingual dietary advice, though performance disparities remain in underrepresented languages such as Kazakh (42). This highlights the need for local dietary data integration and tailored LLM training. Mobile and decentralized implementations, like the SpeziLLM fog-computing framework, offer privacy-aware execution of LLMs for diet-related interventions across healthcare scenarios (43). Studies evaluating GPT-4's analysis of health data also reveal its strength in detail-rich summaries, although expert oversight is essential for ensuring interpretive accuracy (44).

Importantly, a forward-looking research agenda emphasizes personalized food advice as a means to address chronic conditions such as hypertension and allergies through recommender systems (AI tools that suggest personalized options based on user data), along with behavioral modeling and clinical validation (45). However, achieving widespread clinical adoption requires interdisciplinary collaboration, evidence-backed implementation, and transparent model governance. A summary of key AI applications in personalized nutrition, including domains, scientific contributions, and representative references, is provided in Table 2.

**Table 2:** AI applications in personalized nutrition: domains, contributions, and evidence.

**Application domain**	**Scientific contribution**	**Representative references**
Personalized diet planning via multi-omics	Tailors diets using genomic, microbiome, and metabolic data integrated with ML	(11, 12, 14–16)
Dynamic dietary adjustment	RL and DL models personalize recommendations based on metabolic and behavioral feedback	(20, 21, 24–26)
Food image analysis with DL	CNNs and transformers identify food, estimate portion size and nutrients in real time	(29–35)
NLP for dietary coaching	Uses GPT/BERT models to interpret food diaries, support chatbot coaching, and boost adherence	(36–39)
Clinical diagnosis and monitoring	AI models support diet planning for MASLD, and metabolic syndromes; enable explainability	(40–42, 44)
Decentralized and privacy-aware deployment	LLMs deployed on fog computing and mobile devices enhance privacy and local context sensitivity	(43)
Policy, ethics, and interoperability	Addresses equity, algorithmic transparency, language bias, and evidence-based guidance gaps	(27, 28, 45)

#### 2.5.1 Real-world applications of AI in personalized nutrition: the cases of ZOE and DayTwo

AI-powered platforms, such as ZOE and DayTwo, exemplify the practical implementation of personalized nutrition, showcasing how data-driven insights can be harnessed to tailor dietary recommendations at the individual level. *ZOE*, a pioneering startup in precision nutrition, leverages advanced ML algorithms together with comprehensive biological data such as gut microbiome composition, postprandial glycemic responses, and blood lipid profiles to generate individualized dietary recommendations tailored to users' metabolic and physiological responses. By leveraging CGM data alongside microbiota and metabolic biomarkers, ZOE predicts individual responses to different foods in real time and adjusts dietary suggestions accordingly. This holistic and adaptive approach aims to optimize metabolic health and prevent diet-related chronic diseases (46).

In a similar vein, *DayTwo* employs metagenomic sequencing combined with AI-driven predictive modeling to generate individualized meal plans. These plans are specifically designed to minimize glycemic responses in individuals, particularly those with metabolic syndrome, prediabetes, or type 2 diabetes. DayTwo's methodology is grounded in large-scale clinical data and validated through studies demonstrating significant improvements in glycemic control and patient adherence (47). Together, these platforms illustrate how AI technologies are translating the principles of precision nutrition into scalable and clinically relevant tools, enabling more proactive and personalized health interventions.

## 3 Predictive analytics for health optimization

### 3.1 Predictive modeling for nutritional deficiency and disease risk

This methodology leverages predictive modeling to assess nutritional deficiencies and disease risks through the integration of ML and AI, enabling early identification of at-risk individuals and supporting personalized dietary interventions (48).

Data sources and preprocessing: predictive models are built using diverse sources such as electronic health records (EHRs), dietary intake surveys, and genomic data. Preprocessing steps include normalization, imputation of missing values, and encoding of categorical data, ensuring consistent integration across lifestyle, clinical, and genetic variables (49).

Feature selection and model training: feature selection incorporates domain-specific risk models (e.g., QRISK3 for cardiovascular risk in the UK, and SCORE2 for estimating 10-year risk of heart disease in Europe) (48, 50) using methods such as recursive feature elimination and SHapley Additive exPlanations (SHAP) analysis to identify high-impact predictors. Models trained include gradient boosting decision trees (GBDT) and deep neural networks, optimized with frameworks like AutoPrognosis (51), and supported by high-performance libraries such as TensorFlow (52) and PyTorch (53). Batch normalization (54), which accelerates training and improves convergence, and dropout regularization (55), which helps prevent overfitting, are also used to enhance model training stability.

Risk stratification and personalized intervention: individuals are stratified using clustering (e.g., k-means) and quantile binning to generate targeted dietary guidance. Outputs guide interventions addressing common deficiencies, including calcium and vitamin D (48).

Validation and performance metrics: model reliability is validated using external datasets and evaluated with metrics such as AUC-ROC and calibration plots (56, 57). Tools such as Scikit-learn (58) ensure reproducibility and comparability across pipelines. The integration of predictive modeling into nutrition science empowers early intervention and personalized healthcare by translating multidimensional data into actionable dietary strategies. Foundational advances in CNN architectures (59) and contemporary optimization algorithms support robust, scalable implementation across diverse health settings.

### 3.2 Federated learning for privacy-preserving health data analytics

FL has emerged as a key approach to addressing privacy concerns in AI-driven healthcare analytics. Unlike traditional ML methods that require centralized data storage, FL enables decentralized model training by keeping patient data localized while only sharing encrypted model updates. This paradigm is particularly relevant in health and nutrition analytics, where privacy and security are critical concerns (60, 61). Recent advancements in FL frameworks have introduced secure aggregation protocols that protect individual gradients during model updates. For instance, VerifyNet employs a double-masking protocol to ensure that sensitive patient data remains confidential throughout the training process (60). Additionally, FL models incorporating differential privacy and homomorphic encryption techniques have been explored to further enhance security, mitigating risks associated with potential data breaches (62, 63). Consent-based protocols, such as Consent-based Privacy-preserving Decision Tree Evaluation (CPDE), allow for encrypted decision tree evaluations in healthcare services while complying with patient consent requirements (63).

The integration of FL with blockchain technology has been proposed to enhance trust and transparency in decentralized health data analytics. Blockchain-based FL architectures allow immutable record-keeping of model updates without exposing raw health data, supporting applications like cross-domain EHRs sharing and nutrition recommendation systems (61, 64). In smart city health monitoring systems, FL is increasingly combined with Internet of Medical Things (IoMT) to track obesity risk and support tailored interventions (61). Practical implementations of FL have emerged across healthcare education and mental health monitoring. For example, the FAITH project demonstrates a federated AI framework to monitor depressive symptoms in cancer survivors using data from nutrition, sleep, activity, and voice markers via wearable technologies (65). Similarly, the hybrid FL-enabled depression prediction model proposed by 66. Quang Tran and Byeon (66) applies synthetic tabular data from national nutrition surveys and integrates transformer-based models to enhance explainability and privacy. Educational innovations have also adopted federated or privacy-preserving paradigms. Game-based nutrition learning integrated with digital diet assessment tools has shown effectiveness in promoting dietary behavior among adolescents (67), and interprofessional curricula on health promotion, encompassing nutrition, physical activity, mindfulness, and emotional regulation–are increasingly incorporating AI and secure data tools in medical and nursing education (68).

Despite its advantages, FL presents challenges including computational overhead, communication efficiency, and regulatory compliance. The ethical and legal implications of decentralized AI in healthcare necessitate robust governance frameworks. Researchers emphasize the importance of privacy-preserving techniques in AI governance to ensure alignment with General Data Protection Regulation (GDPR) and Health Insurance Portability and Accountability Act (HIPAA) regulations (62). The application of hyperparameter-tuned ML models in youth health monitoring has yielded high classification accuracy for physical fitness assessment, highlighting the parallel need for robust ethical standards in educational data practices (69). As AI-driven health analytics continue to evolve, FL remains a promising solution for enabling secure and privacy-preserving data analysis. Future research directions include refining privacy-enhancing techniques, optimizing computational efficiency, and ensuring that FL-based systems comply with ethical and legal standards in healthcare and nutrition applications (60, 65, 69).

### 3.3 AI-driven strategies for chronic disease prevention and management

The growing prevalence of chronic diseases such as diabetes, obesity, and cardiovascular conditions necessitates novel strategies that combine AI with digital health innovations. AI has proven effective in generating adaptive nutrition plans, enhancing self-management, and supporting decision-making in clinical and community settings (70–72).

AI-driven systems are increasingly used to monitor physiological data and personalize care. Tools like AI-based telemedicine platforms and electrochemical breathomics sensors allow continuous monitoring of patient metrics, e.g., glucose, dietary intake, and respiratory biomarkers, thereby improving the management of diseases such as COPD, CKD, and diabetes (73, 74). Breathomics coupled with AI further enables early diagnosis through noninvasive volatile organic compound analysis, demonstrating potential in chronic respiratory and renal disease detection (73). In diabetes care, AI tools assist in clinical risk assessment, glycemic control, and public health decision-making, particularly in underserved regions (74). AI-integrated education tools such as ChatGPT have also shown promise in nursing and dermatology settings by supporting clinical reasoning and training in high-stakes environments (75, 76). Similarly, game-based learning and VR-enhanced self-directed education for students have improved health literacy, nutritional awareness, and disease preparedness (77), highlighting AI's integrative role in patient care and public health initiatives (see Table 3).

**Table 3:** AI applications in chronic disease prevention and management: domains, contributions, and evidence.

**Application domain**	**Scientific contribution**	**Representative references**
Adaptive nutrition and metabolic control	AI-driven tools personalize diet plans, track glucose levels, and optimize interventions for obesity and diabetes	(70–72, 74)
Respiratory and renal diagnostics	AI-integrated breathomics enables noninvasive VOC-based early detection for COPD, CKD, and lung disease	(73)
AI-enabled education	ChatGPT supports decision-making and clinical training in clinical practice	(75, 76)
Gamified health literacy	VR-enhanced, ML-guided education boosts student awareness on nutrition, sustainability, and chronic risk	(77)
Risk stratification and youth monitoring	ML predicts water usage behavior, fitness level, and foresight on public health challenges	(69, 78, 79)
FL for IoMT health systems	Distributed FL systems support privacy-preserving monitoring for obesity and cancer survivorship across health networks	(61, 65)
AI in medicinal plant therapeutics	AI and bioinformatics identify active plant compounds and support personalized herbal medicine	(80)
Immunological precision care	AI supports allergen prediction, immune profiling, and targeted interventions in allergy/immunology	(81)
Ethics and explainability in healthcare AI	LLMs used in clinical practice and patient education raise concerns on algorithmic bias and data transparency	(76, 82, 131)

ML applications have also enabled risk prediction and behavior analysis related to water sustainability, fitness classification, and foresight on global health challenges among students (69, 78, 79). Furthermore, FL frameworks offer scalable, privacy-preserving solutions for chronic disease monitoring across distributed healthcare networks, as exemplified in cancer survivorship studies and obesity management using IoMT devices (61, 65). The convergence of AI, ML, and bioinformatics also facilitates compound identification in medicinal research, supporting the development of plant-based therapeutics and personalized treatment strategies (80). These tools extend to allergy and immunology, where AI supports allergen prediction, immune profiling, and targeted interventions (81).

Despite these advances, challenges persist. Ethical concerns around data privacy, interpretability of models, and algorithmic bias require transparent, secure, and patient-centered design frameworks (76, 82). To ensure the sustainability and efficacy of AI in chronic disease management, future research must focus on long-term clinical outcomes, adaptability to diverse populations, and the integration of AI into regulatory and educational infrastructures.

#### 3.3.1 Real-world application: nutrino health's AI-powered solutions for diabetes management

*Nutrino Health*, a company in AI-driven nutrition analytics, developed predictive models that integrate data from CGM systems, dietary intake logs, and individual health profiles to forecast personalized glycemic responses. By analyzing the complex interplay between food intake and glucose fluctuations, Nutrino's algorithms provide insights into how specific meals affect blood sugar levels in real-world settings. This personalized predictive approach enables more effective meal planning and glucose control, especially for individuals managing diabetes. The clinical utility of Nutrino's technology attracted significant interest, leading to its acquisition by Medtronic. The integration of Nutrino's analytics into Medtronic's diabetes management platforms aimed to enhance closed-loop insulin delivery systems and provide real-time, tailored dietary guidance for patients. This case exemplifies how AI-based nutritional modeling can be translated into tangible clinical tools, supporting both improved therapeutic outcomes and patient self-management (83).

## 4 AI-driven innovations in food manufacturing

### 4.1 AI in smart food production

The integration of AI technologies in food manufacturing is transforming traditional practices by enhancing efficiency, quality assurance, and sustainability (84). AI-driven automation supports predictive decision-making, streamlines process workflows, and minimizes operational waste. For instance, Kumar et al. (85) demonstrate that ML models can optimize ingredient mixing, energy usage, and production parameters. Similarly, Misra et al. (86) highlight the role of AI, IoT, and big data analytics in enabling intelligent, responsive decision-making across agri-food systems.

Significant advancements in supervised learning and machine vision have improved quality control. Zhu et al. (87) report the successful deployment of CNNs for real-time defect detection, increasing both accuracy and consistency. Cognitive cloud robotics, as discussed by Wan et al. (88), further enhances logistical planning and energy efficiency in food plants. However, as Sarker et al. (89) caution, the increasing reliance on interconnected AI systems elevates cybersecurity risks, necessitating robust frameworks to safeguard food manufacturing infrastructure. A summary of AI applications across production automation, quality control, inventory, and traceability is provided in Table 4.

**Table 4:** AI applications in food manufacturing: domains, innovations, and impact.

**Application domain**	**Technological contributions**	**Representative references**
Smart production automation	Optimization of mixing, energy, and logistics using ML and robotics	(85, 86, 88)
Machine vision for quality control	Real-time defect detection and image-based contamination screening	(87, 98, 99)
Inventory management	AI-based demand forecasting and smart packaging for perishables	(91, 92)
Predictive maintenance	Condition monitoring and failure prediction via ML and DL models	(93–95)
Waste management	AI-assisted composting and anaerobic digestion systems	(96, 97)
Food safety and traceability	AI with blockchain for tracking, quality assurance, and compliance	(102–104)

#### 4.1.1 Real-world application: Timestrip^Ⓡ^ smart indicators in intelligent food packaging

*Timestrip*^Ⓡ^ is a widely adopted smart packaging solution that offers visual indicators for tracking elapsed time and temperature exposure, particularly in cold-chain logistics. These indicators change color or display a visual signal once a product has been exposed to conditions that may compromise its safety or quality. Originally developed for simplicity and low-cost implementation, Timestrip^Ⓡ^ devices have become a foundational technology in supply chain monitoring. When integrated with IoT infrastructures and AI-driven analytics, Timestrip^Ⓡ^ solutions evolve beyond passive indicators. In smart packaging ecosystems, these indicators serve as real-time data sources, transmitting information on environmental conditions to centralized platforms. AI models can analyze this data to predict spoilage risk, estimate remaining shelf life, and dynamically optimize storage and transportation strategies. This integration enables food manufacturers, retailers, and logistics providers to enhance product safety, minimize waste, and ensure compliance with temperature-sensitive regulations (90).

### 4.2 Waste reduction and resource optimization

AI plays a crucial role in advancing sustainability goals in food manufacturing by improving inventory management, enabling predictive maintenance, and supporting environmentally conscious waste treatment strategies.

#### 4.2.1 AI-powered inventory management for food waste reduction

AI-based inventory management systems are transforming food supply chains by enhancing efficiency and reducing waste. Through real-time demand forecasting, AI algorithms are capable of accurately predicting consumption trends, thereby minimizing overproduction and spoilage (91). In addition, sensor-enabled smart packaging, combined with AI analytics, enables continuous monitoring of product freshness and shelf life. This facilitates better-informed storage and logistics decisions, significantly reducing the chances of inventory expiration (92). Furthermore, data-driven models empower dynamic stock control mechanisms, ensuring timely rotation and efficient allocation of perishable goods. These innovations collectively support sustainable inventory practices while reducing operational and environmental costs.

#### 4.2.2 Predictive maintenance for sustainable production

Predictive maintenance frameworks, empowered by AI, play a crucial role in advancing sustainable food production. ML models are adept at detecting early signs of equipment malfunction by identifying subtle deviations in operational data, thus enabling timely interventions before costly breakdowns occur (93, 94). Moreover, AI-driven algorithms help optimize maintenance schedules by predicting the optimal time for service and repair, which prolongs equipment lifespan and ensures consistent production efficiency (95). These strategies contribute to minimizing unplanned downtime and conserving resources across manufacturing operations.

#### 4.2.3 Waste management and environmental sustainability

AI technologies are increasingly aligned with circular economy principles, offering advanced solutions for waste reduction and environmental sustainability in food manufacturing. Intelligent systems enhance composting efficiency by improving the classification of organic waste and enabling real-time monitoring of decomposition processes (96). Additionally, AI supports anaerobic digestion by optimizing operational parameters, leading to more efficient bioenergy production. This not only helps reduce greenhouse gas emissions but also facilitates the conversion of food waste into renewable energy resources (97). These applications underscore AI's role in promoting eco-friendly waste treatment and resource recovery strategies.

### 4.3 Quality control and food safety with AI

AI technologies are transforming food safety and quality control by introducing automation, precision, and enhanced traceability across the food supply chain. Advanced machine vision systems, driven by AI algorithms, are now capable of identifying microbial and fungal contaminants in both raw ingredients and finished food products, enabling early detection and mitigating the risk of foodborne outbreaks (98, 99). Furthermore, AI has been employed to optimize natural preservation strategies–for example, by enhancing the performance of lactic acid bacteria used as bio-preservatives, thereby extending shelf life while maintaining safety (100). The integration of metabolomic profiling with predictive AI models further supports proactive food safety management by identifying early spoilage indicators and physiological markers of contamination (101). In addition, the convergence of AI and blockchain technologies has given rise to comprehensive traceability systems that automate regulatory compliance, ensure supply chain transparency, and rapidly pinpoint sources of contamination (102–104).

#### 4.3.1 Real-world application: blockchain-enabled food traceability through IBM food trust and TE-FOOD

Blockchain technology has emerged as a transformative tool in the realm of food traceability, offering transparency, data immutability, and real-time access across the supply chain. *IBM Food Trust*, one of the most prominent blockchain platforms in this space, enables end-to-end traceability of food products by securely recording transactions and movements from farm to shelf. Major global retailers such as Walmart and Carrefour have adopted the system to rapidly identify sources of foodborne illness, authenticate the origins of products, and streamline recall processes. By digitizing each step in the food supply chain, IBM Food Trust enhances accountability, reduces response times in food safety incidents, and builds consumer trust (105).

Complementing this, *TE-FOOD* offers blockchain-based solutions specifically tailored for the traceability of livestock and agricultural produce. Operating in both developed and emerging markets, TE-FOOD integrates digital identification, GPS tracking, and mobile data collection to ensure food safety, prevent fraud, and comply with regulatory frameworks. Its implementation in developing countries has been especially impactful, supporting local authorities and producers in building more transparent and efficient food systems (106, 107). Together, platforms like IBM Food Trust and TE-FOOD exemplify how blockchain can reinforce resilience, integrity, and sustainability in global food supply networks by creating tamper-proof records and fostering stakeholder collaboration across complex distribution channels.

## 5 Interdisciplinary collaboration in nutrition and AI research

**Figure 1:**
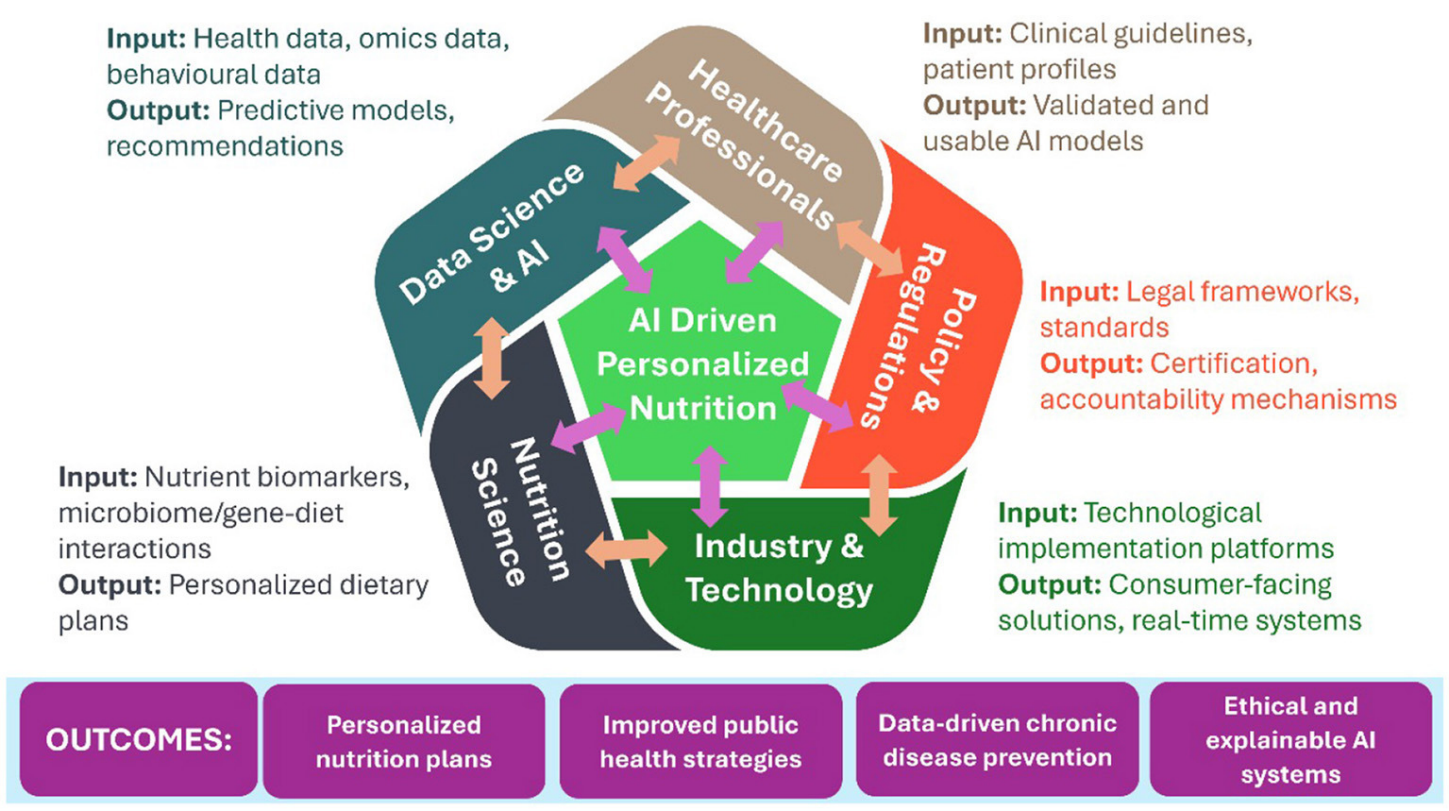
A conceptual framework illustrating interdisciplinary collaboration in AI-driven personalized nutrition. The model integrates contributions from data science, healthcare, nutrition, industry, and policy to produce ethically grounded, clinically valid, and context-sensitive dietary solutions.

The integration of AI into the field of nutrition science necessitates robust interdisciplinary collaboration, particularly between data scientists, healthcare professionals, and nutrition experts. Data scientists offer sophisticated analytical tools capable of processing high-dimensional, heterogeneous datasets, while clinicians and dietitians ensure that these AI-driven systems are grounded in medical relevance, ethical soundness, and adherence to evidence-based nutritional guidelines (4, 5). This cross-disciplinary synergy is essential for advancing precision nutrition paradigms that reflect inter-individual variability in genetic makeup, lifestyle factors, and health status. In parallel, the ethical implementation of AI in personalized nutrition calls for the incorporation of privacy-preserving mechanisms such as FL and homomorphic encryption. These techniques are critical for safeguarding sensitive personal and clinical data, particularly in decentralized or multi-institutional healthcare environments. Beyond data security, promoting transparency and interpretability in AI model outputs is vital to building trust among end-users, clinicians, and regulatory bodies (108, 109). Figure 1 represents a conceptual framework for interdisciplinary collaboration in the AI and Personalized Nutrition landscape

Substantial progress has also been observed in the application of AI to genomics and microbiome research. In nutrigenomics, ML models are increasingly employed to elucidate complex gene-diet interactions, providing a foundation for the development of tailored dietary recommendations. Concurrently, the use of AI in microbiome science has facilitated a deeper understanding of host-microbiota dynamics and their implications for metabolic and immunological health (5, 47). DL architectures, such as CNNs, are particularly valuable in decoding genomic and microbial signatures that serve as biomarkers of nutritional responsiveness (4). These technologies enhance both the predictive accuracy and scalability of biomarker discovery, advancing the clinical applicability of personalized nutrition strategies. To further accelerate innovation and translational impact, partnerships between academia and the health and food industries have become increasingly prominent. These collaborations support the development of functional foods, nutraceuticals, and AI-enabled platforms for dietary assessment and personalized recommendation. For example, industry-academic consortia have pioneered smart packaging technologies capable of real-time quality monitoring, thereby reducing food spoilage and ensuring safety across the supply chain (91, 110). Such innovations exemplify the practical deployment of AI-informed systems in both consumer and clinical nutrition contexts.

Finally, the successful implementation of AI technologies in nutrition and healthcare demands the establishment of comprehensive policy frameworks and regulatory governance structures. Public policy must address concerns related to algorithmic fairness, transparency, and the clinical validation of AI tools. Regulatory harmonization across sectors can facilitate the standardization of AI practices and enhance public confidence in digital health interventions (62). Moreover, government agencies play a strategic role in funding and supporting interdisciplinary research initiatives that bridge AI, nutrition, and public health. These efforts are particularly crucial in developing scalable, evidence-based solutions for managing malnutrition, obesity, and other chronic conditions through individualized dietary interventions (48).

## 6 Ethical considerations and challenges

As AI transforms nutrition and food manufacturing, it brings forth a spectrum of ethical considerations, ranging from the protection of personal data and equitable algorithmic design to workforce implications and ecological sustainability. Addressing these challenges is essential to ensure that AI adoption advances public health, equity, and sustainability.

### 6.1 Data privacy and security in AI-driven nutrition

Managing sensitive health and dietary data remains a core concern in AI-based nutrition systems. Given the deeply personal nature of such data, ensuring robust safeguards against breaches and misuse is imperative. Differential privacy, FL, and homomorphic encryption have emerged as key strategies to protect user data during model training without compromising analytical performance (111, 112). Compliance with regulatory frameworks such as the GDPR and HIPAA is necessary to uphold ethical standards in AI deployment. However, despite these innovations, data breaches and adversarial vulnerabilities persist. Aldoseri et al. (113) emphasize the need for domain-specific data protection strategies tailored to the unique risks posed by dietary and health datasets. Moving forward, the development of user-centric privacy frameworks that promote transparency and informed consent will be crucial.

### 6.2 Bias and fairness in AI nutrition models

Bias embedded in training datasets and algorithmic design can lead to unequal access and skewed health recommendations, particularly for underserved or culturally diverse populations. As Saraswat et al. (114) point out, models trained on homogenous or Western-centric data may fail to generalize across socio-economic or ethnic groups. Zhao and Chen (112) suggest differential privacy as a mechanism to improve demographic representativeness during training. Nonetheless, there remains a critical need for standardized evaluation frameworks to assess fairness and inclusivity in AI-generated nutrition advice. Research should also prioritize the inclusion of culturally relevant dietary patterns and genetic diversity in model development.

### 6.3 Ethical dilemmas in AI-driven food manufacturing

The deployment of AI in food manufacturing introduces socio-ethical trade-offs, especially concerning labor displacement and transparency in decision-making. While AI-driven automation improves efficiency and reduces operational costs, it may also threaten the livelihoods of manual workers. Bartoletti (115) and Himeur et al. (116) call for strategies that incorporate human-AI collaboration rather than substitution. Additionally, ensuring interpretability of AI decision processes in manufacturing settings is vital for maintaining stakeholder accountability. Ethical implementation requires proactive reskilling programs, inclusive workforce policies, and explainable AI frameworks that demystify decision-making processes.

### 6.4 Sustainability and environmental ethics in AI deployment

AI can significantly advance sustainability in food systems through precision manufacturing, waste reduction, and supply chain optimization. However, the energy demands of AI, particularly DL, raise concerns about their ecological footprint. Režek Jambrak et al. (117) caution against overlooking the environmental costs of training large-scale AI models. In contrast, Selvarajan et al. (118) emphasize the potential for AI to contribute to net-positive sustainability outcomes through efficient resource management. Agrawal et al. (119) also highlight AI's transformative potential for sustainability in food manufacturing by integrating circular economy practices and minimizing environmental externalities. A careful balance must be maintained between AI's resource consumption and its capacity to drive sustainable practices.

Furthermore, the ethical use of AI in dietary interventions for vulnerable populations must consider access, digital literacy, and equity. Kalyoncu Atasoy et al. (120) highlights the importance of developing inclusive AI-powered nutrition strategies that are sensitive to the needs of at-risk groups. The ethical deployment of AI in nutrition and food systems necessitates a multi-stakeholder approach that integrates regulatory oversight, interdisciplinary collaboration, and inclusive societal engagement. Recent contributions have also emphasized the necessity of transparent, explainable, and socially accountable AI frameworks in high-stakes environments such as healthcare (108, 109).

## 7 Future perspectives and innovations

**Figure 2:**
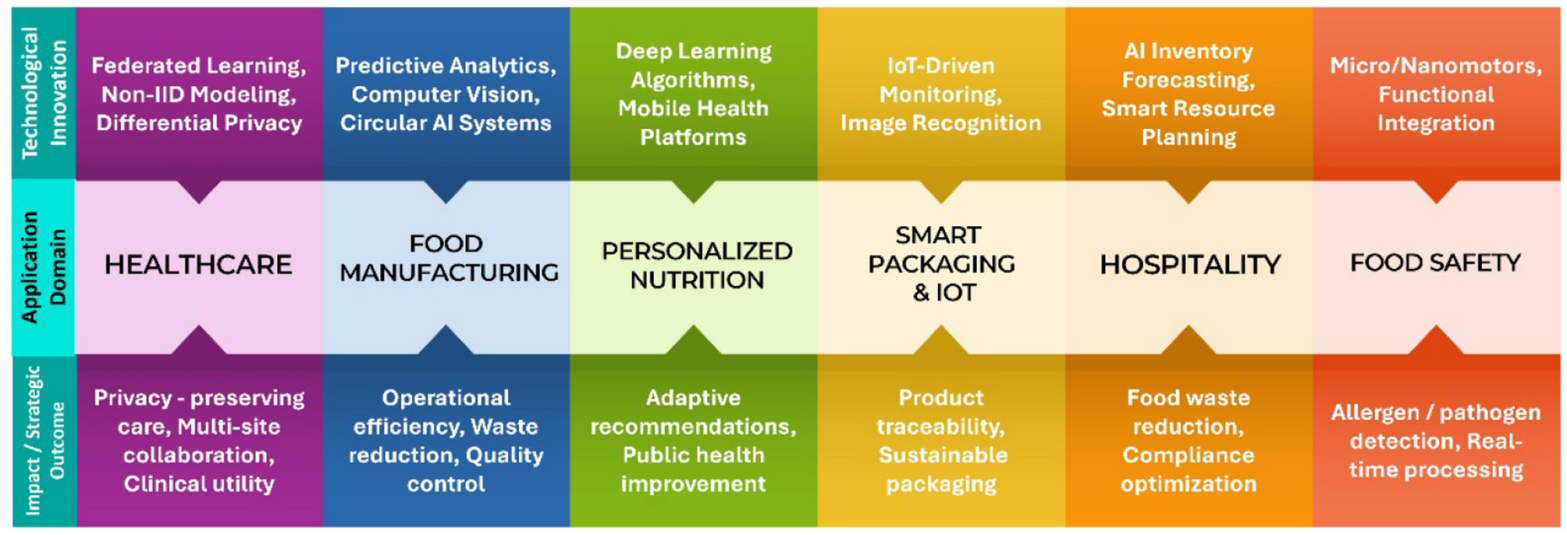
Conceptual mapping of emerging innovation trajectories across key application domains. The figure illustrates how advanced technologies such as federated learning, IoT, and AI-driven systems intersect with healthcare, food production, packaging, and hospitality, enabling targeted outcomes in privacy, sustainability, and efficiency.

The intersection of FL, AI, IoT, and sustainability presents transformative opportunities across healthcare, food systems, and hospitality sectors. Emerging directions focus on privacy-respecting data ecosystems, intelligent automation, and environmentally conscious design principles. Key thematic advancements are outlined below and also in Figure 2.

- FL in healthcare: next-generation FL frameworks address challenges of data heterogeneity through non-IID degree estimation (121), incorporate adaptive regularization, and integrate differential privacy mechanisms for clinical applicability (122, 123). These systems promote collaborative, secure, and equitable healthcare innovation.
- AI in food manufacturing: AI applications in food processing increasingly support real-time optimization, predictive quality control, and defect detection. Integration with circular economy models enhances resource efficiency and aligns manufacturing with sustainable development goals (119, 124).
- Personalized nutrition technologies: ML-driven mobile platforms deliver context-aware dietary recommendations tailored to physiological, behavioral, and demographic profiles. Such systems enhance maternal health and broader public health outcomes through scalable, user-centric interventions (125, 126).
- Smart packaging and IoT integration: intelligent packaging incorporates IoT connectivity, AI-based image analysis, and entropy-based design optimization to improve tracking, labeling, and freshness monitoring (127, 128). Emphasis shifts toward sustainable packaging materials and real-time data flow.
- Sustainable hospitality systems: AI-enhanced food waste monitoring and predictive inventory management systems enable eco-efficient hospitality operations while facilitating compliance with regulatory standards (129).
- Micro/nanomotors in food safety: functionalized micro/nanomotors emerge as versatile tools for pathogen detection, allergen removal, and sterilization. Research increasingly focuses on cost-effective fabrication, multifunctional integration, and biocompatibility for safe industrial deployment (130).

Current trends emphasize ethical data handling, intelligent automation, and eco-innovation. This section outlines the core areas of advancement and anticipated directions, with a synthesis presented in Table 5.

**Table 5:** Emerging innovation trajectories across key domains: a technological focus.

**Innovation area**	**Future perspective**	**Key references**
FL in healthcare	Robust architectures incorporate non-IID adaptation, privacy-preserving protocols, and multi-institutional scalability.	(121–123)
AI in food manufacturing	Circular economy-aligned systems enable real-time optimization, quality control, and waste reduction.	(119, 124)
Personalized nutrition	AI-driven mobile platforms offer adaptive and personalized nutrition interventions.	(125, 126)
Smart packaging and IoT	IoT-enabled packaging integrates real-time monitoring, digital labeling, and sustainability.	(127, 128)
Sustainable hospitality	AI tools support food waste tracking and operational efficiency in hospitality environments.	(129)
Micro/nanomotors in food safety	Micro/nanomotor systems address food safety diagnostics and functional processing tasks.	(130)

## 8 Conclusion

The convergence of AI, personalized nutrition, and intelligent food manufacturing marks a paradigm shift in how health and food systems operate. By leveraging ML, DL, and FL, AI transforms both dietary planning and production workflows into adaptive, data-driven ecosystems. These technologies enable the real-time delivery of individualized nutritional guidance while also ensuring sustainable, transparent, and optimized food production. This study contributes to the field by offering a comprehensive synthesis of AI applications across personalized nutrition and food manufacturing, identifying key enablers, practical use cases, and emerging research trajectories.

Findings from this comprehensive study highlight that AI has the potential to:

- Deliver individualized nutrition recommendations through multi-omics integration and behavioral modeling.
- Support ethical and privacy-preserving data use via FL and secure analytics frameworks.
- Enable predictive health risk stratification and early dietary intervention for chronic disease prevention.
- Optimize food processing for quality retention, waste reduction, and resource efficiency.
- Strengthen supply chain transparency through AI-driven traceability and smart packaging.

AI's integration into nutrition science and food manufacturing holds transformative promise for public health and sustainability. However, its success depends on addressing pressing challenges, such as data bias, regulatory gaps, model explainability, and digital inequity. Therefore, this study highlights the need for interdisciplinary collaboration among nutritionists, AI researchers, clinicians, and policymakers to establish ethical, evidence-based, and culturally inclusive AI frameworks. Through multi-stakeholder collaboration and a commitment to responsible innovation, the food and nutrition sector can harness the full potential of AI to build resilient, equitable, and personalized health ecosystems. The insights provided in this study lay the foundation for future advancements in research, clinical integration, and sustainable industrial transformation.

## Glossary

- AI (artificial intelligence): the simulation of human cognitive functions such as learning, reasoning, and problem-solving by machines.
- FL (federated learning): a privacy-preserving machine learning approach where decentralized data sources collaboratively train a shared model without exchanging raw data.
- IoT (internet of things): a system of interconnected physical devices that collect and transmit data via the internet to enhance decision-making and automation.
- IoMT (internet of medical things): a subset of IoT encompassing medical devices and health systems connected to networks for real-time data exchange and monitoring.
- ML (machine learning): a branch of AI that enables systems to learn patterns from data and make predictions or decisions without being explicitly programmed.
- DL (deep learning): a subset of ML that uses multilayered neural networks to analyze complex patterns and high-dimensional data.
- CNNs (convolutional neural networks): a type of deep learning model highly effective for image recognition, classification, and feature extraction.
- NLP (natural language processing): an AI subfield enabling computers to interpret, generate, and respond to human language in a meaningful way.
- GANs (generative adversarial networks): a class of ML models composed of two neural networks, generator and discriminator, competing to produce highly realistic synthetic data.
- Explainable AI (XAI): A framework of AI techniques designed to make AI decision-making processes transparent, interpretable, and understandable to humans.
- Blockchain: a decentralized, immutable digital ledger used to securely record transactions, often employed in food traceability and supply chain integrity.
- Smart packaging: packaging embedded with sensors or indicators to provide information on the quality, safety, and condition of the food product.
- Edge computing: a distributed computing paradigm that processes data near the source of generation, improving response times and reducing bandwidth.
- Big data: large, complex datasets that require advanced tools and techniques for analysis, often used in personalized nutrition and food safety analytics.
- Bioinformatics: an interdisciplinary field combining biology, computer science, and statistics to analyze and interpret biological data, particularly in genomics and metabolomics.
- Personalized nutrition: a tailored approach to dietary recommendations based on individual characteristics such as genetic profile, lifestyle, microbiome, and biomarkers.
- Precision health: a broader concept that integrates personalized data to prevent, diagnose, and treat diseases at an individual level.
- Sensor fusion: the integration of data from multiple sensors to improve accuracy and reliability of information in monitoring systems.
- Digital twin: a virtual representation of a physical system (e.g., production facility or human body) used for simulation, prediction, and optimization.
- Sustainable food systems: food production and distribution practices designed to minimize environmental impact while ensuring nutritional adequacy and food security.
- Ethical AI: the practice of designing, deploying, and managing AI systems in a manner that upholds fairness, transparency, privacy, and accountability.
